# African swine fever virus pB318L suppresses inflammatory response by inhibiting NF-κB activation and NLRP3 inflammasome formation

**DOI:** 10.1371/journal.ppat.1013558

**Published:** 2025-10-22

**Authors:** Xiaohong Liu, Guangqiang Ye, Yi Zeng, Hanyu Wu, Siqi Dong, Xiaoping He, Qiongqiong Zhou, Hongyang Liu, Zhaoxia Zhang, Jiangnan Li, Changjiang Weng, Li Huang

**Affiliations:** 1 National African Swine Fever Para-reference Laboratory, State Key Laboratory of Animal Disease Prevention and Control, Harbin Veterinary Research Institute, Chinese Academy of Agricultural Sciences, Harbin, China; 2 Heilongjiang Provincial Key Laboratory of Veterinary Immunology, Harbin, China; Pirbright Institute, UNITED KINGDOM OF GREAT BRITAIN AND NORTHERN IRELAND

## Abstract

African swine fever (ASF) is an acute, hemorrhagic, and severe infectious disease caused by African swine fever virus (ASFV), posing significant threats to global swine production. ASFV pathogenesis is closely associated with its sophisticated immune evasion strategies. In this study, we demonstrate that ASFV pB318L, a trans-geranylgeranyl-diphosphate synthase (GGPPS) homolog inhibited both the NF-κB signaling pathway and the formation of the NLRP3 inflammasome. Infection with ASFV-intB318L (a recombinant ASFV with pB318L expression inhibition) induced significantly higher levels of IL-1β compared to its parent strain ASFV HLJ/18. Mechanically, pB318L interacts with NEMO to inhibit the interaction between IKKα and NEMO, and suppresses the K63-linked ubiquitination of NEMO mediated by TRIM21. In addition, pB318L interacts with the NACHT and LRR domains of NLRP3, which prevents the oligomerization of NLRP3 by suppressing the interaction between NEK7 and NLRP3. Crucially, the immunosuppressive functions of pB318L on both NF-κB signaling pathway and NLRP3 inflammasome activation are independent of its GGPPS enzymatic activity. In conclusion, we presented evidence that ASFV pB318L negatively regulates NF-κB signaling pathway and NLRP3 inflammasome. This study provides critical mechanistic insights into the role of pB318L in ASFV pathogenesis and highlights its potential as a target for the development of antiviral strategies or live-attenuated vaccines against ASF.

## Author summary

ASF is an acute hemorrhagic infectious disease caused by ASFV, which has a serious impact on the global pig industry. The importance of this article lies in revealing the crucial role of ASFV pB318L in viral infection, particularly in how it evades the host’s innate immune response by inhibiting the activation of the NF-κB signaling pathway and NLRP3 inflammasome. This discovery not only deepens our understanding of the biological characteristics of ASFV, but also provides new research clues for the development of antiviral drugs or live vaccines against ASF. By investigating the interaction between pB318L and NEMO, as well as pB318L and NLRP3, this article provides a critical mechanistic explanation of how ASFV promotes its own survival by modulating host immune mechanisms. In addition, these findings lay the foundation for the prevention and control strategies of ASF in the future, especially in the context of the increasingly urgent global need to manage ASF outbreaks. Therefore, the research results of this study hold important theoretical and practical implication for fields such as public health, animal disease prevention and control, and agricultural economics.

## Introduction

African swine fever (ASF) is an acute and highly contagious disease caused by African swine fever virus (ASFV), which is listed as a notifiable animal disease by the World Organization for Animal Health (WOAH) [1]. ASF outbreaks were first reported in Kenya in 1921 and have since spread widely and erupted on a large scale in Africa. In 2007, ASFV was introduced from Africa to the Caucasus region, and in 2014, it had spread significantly throughout Eastern Europe [2]. In August 2018, China reported its first outbreak of ASF to the WOAH, making the initial occurrence of this highly contagious viral disease in East Asian swine populations [3]. ASFV primarily infects domestic pigs and wild boars, causing a range of clinical manifestations. Highly virulent strains can lead to death in infected pigs within 4–15 days, with mortality rates approaching 100% [1]. Currently, ASF poses a significant threat globally, causing substantial economic and ecological impacts.

ASFV is the sole member of the Asfivirus genus within the Asfarviridae family. The virus particle has a diameter of about 260 nm, with an icosahedral symmetry capsid structure. The genome of ASFV is a double-stranded linear DNA, ranging from 170 to 193 kb, containing 150–167 ORFs and encoding 165 and above viral proteins [4]. The viral particle is composed of multiple structural layers, from inside to outside: the viral genome, the nuclear capsid, the inner membrane, the capsid, and the envelope [5]. Beyond encoding proteins essential for viral replication and infection, ASFV encodes an array of immunomodulatory proteins that antagonize the host’s antiviral innate immune response, including inhibiting interferon (IFN) production, regulating inflammation responses, and manipulation of host cell processes such as apoptosis, autophagy, as well as host cell protein synthesis [6].

During viral infection, the inflammatory cytokines produced by the host play a critical role in antiviral defense. The cytokines not only directly inhibit viral replication, but also orchestrate adaptive immune responses that facilitate the elimination of infected cells via cytotoxic lymphocyte mechanisms [7]. The activation of inflammatory response requires two separate signals. Signal 1, nuclear factor-kappa B (NF-κB) pathway, can be activated by both extracellular and intracellular stimuli. Extracellular activation occurs when specific ligands bind to cell surface receptors, initiating a downstream signaling cascade. Additionally, intracellular stimuli, such as pathogen-associated molecular patterns (PAMPs) generated during viral infection can also induce NF-κB activation. Upon stimulation, receptor proteins activate IκB kinase (IKK), which subsequently phosphorylates the serine residues at the regulatory site of the IκB subunit within the intracellular NF-κB/IκB complex. The phosphorylation event marks the IκB subunit for ubiquitination and degradation by proteasomes, thereby releasing NF-κB dimers [8,9]. The liberated NF-κB translocates to the nucleus and binds to NF-κB-reponsive elements, promoting the transcription of NOD-like receptor family pyrin domain containing 3 (NLRP3) and interleukin-1β (IL-1β) [9]. Signal 2 is associated with the activation of various inflammasomes, including NLRP3, NLR family CARD domain containing 4 (NLRC4), and absent in melanoma 2 (AIM2), which collectively activate caspase-1 [10]. Once activated, caspase-1 cleaves pro-IL-1β, pro-IL-18, and gasdermin D (GSDMD), leading to the maturation and secretion of IL-1β and IL-18 through pyroptosis [11].

ASFV pB318L is encoded by the late ASFV gene *B318L* and consists of 318 amino acids. It exhibits homology to geranylgeranyl-diphosphate synthase (GGPPS) [12,13], sharing four highly conserved amino acid regions that are unique to these enzymes. So far, the ASFV *B318L* gene is the only identified viral-origin prenyltransferase-related gene [12]. Moreover, pB318L is expressed during the late stage of viral morphogenesis, suggesting that its function may be necessary for viral particle assembly and release [14]. Our previous research has demonstrated that ASFV pB318L plays a role in antagonizing type I IFN production and response. Additionally, pB318L inhibits the NF-κB promoter activity induced by the cGAS-STING pathway [15], which suggests that ASFV pB318L may plays a role in inhibiting host inflammatory responses.

In this study, the mechanisms of ASFV pB318L modulating host inflammatory responses were elucidated. We found that ASFV pB318L effectively suppresses the inflammation response through inhibiting the activation of NF-κB signaling and the formation of NLRP3 inflammasome. Specifically, pB318L inhibits NF-κB signaling pathway through targeting NEMO (NF-κB Essential Modulator), and it interacts with NEMO, thereby disrupting the interaction between IKKα and NEMO, and preventing K63-linked ubiquitination of NEMO. Additionally, pB318L also suppresses the interaction between NIMA-related kinase 7 (NEK7) and NLRP3 by directly interacting with NLRP3, thereby inhibiting the oligomerization of NLRP3. Furthermore, pigs infected with ASFV-intB318L (an ASFV strain with inhibited pB318L expression) exhibited significantly higher levels of inflammatory cytokines compared to those infected with wild-type ASFV (ASFV-WT). In summary, our research indicated that the ASFV *B318L* gene is a potent immunosuppressive gene. It not only antagonizes type I IFN responses but also effectively inhibiting the inflammatory response process.

## Results

### ASFV pB318L inhibits NF-κB signaling pathway

**Fig 1:**
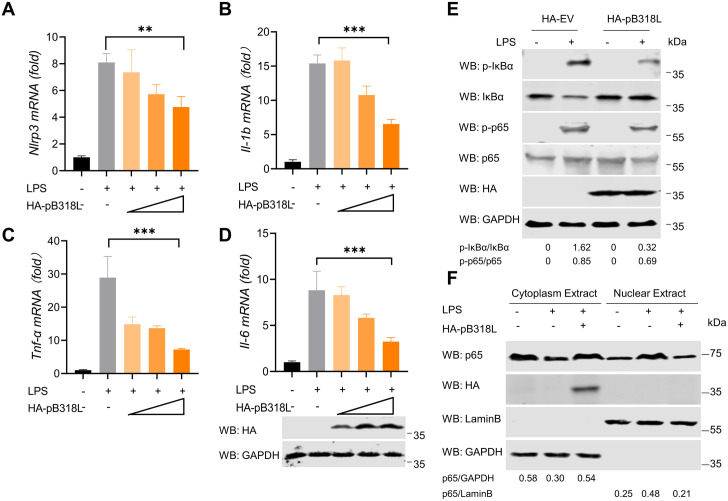
Ectopic expression of pB318L inhibits the NF-κB signaling pathway. **(A-D)** HeLa cells were transfected with a plasmid expressing HA-pB318L for 24 h and stimulated with LPS for another 6 h, and then the mRNA levels of **Nlrp3, Il-1b, Tnf*α,* and *Il-6* were detected by qPCR. **(E)** HeLa cells were transfected with a plasmid expressing HA-pB318L or empty vector for 24 h and then stimulated with LPS for 1 h. The expression and phosphorylation of p65 and IκB were detected by Western blotting. Grayscale values were quantified using ImageJ. **(F)** HeLa cells were transfected with a plasmid expressing HA-pB318L for 24 h and stimulated with LPS for 6 h. The cells were harvested and the distribution of p65 was detected. Grayscale values were quantified using ImageJ. Data are representative of three independent experiments with three biological replicates (mean ± s.d.). Ns, not significantly, ** **p* *< 0.01, *** *p* < 0.001, (one-way ANOVA).

To confirm whether pB318L inhibits the NF-κB signaling pathway, HeLa cells were transfected with a plasmid expressing pB318L and then stimulated with lipopolysaccharide (LPS). The results showed that overexpression of pB318L inhibited LPS-induced mRNA levels of *Nlrp3*, *Il-1b*, Tumor Necrosis Factor-α (*Tnf-α*), and *Il-6* in a dose dependent manner (Fig 1A–1D). Consistent with these results, overexpression of pB318L also inhibited the phosphorylation of IκB and p65 (Fig 1E). Given that p65 forms a dimer with p50 and subsequent translocates to the nucleus, and inducing the expression of pro-IL-β. Therefore, the effect of pB318L on the translocation of p65 to the nucleus was examined. Western blotting analysis revealed that pB318L inhibited the LPS-induced nuclear localization of p65 (Fig 1F). The above results indicate that the exogenous expression of pB318L inhibits the activation of the NF-κB signaling pathway.

**Fig 2:**
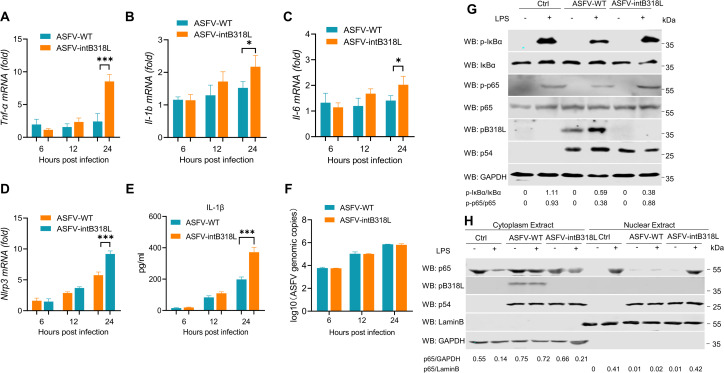
ASFV pB318L inhibits NF-κB upon ASFV infection. **(A-F)** PAMs were mock infected or infected with ASFV-WT or ASFV-intB318L for 6, 12, or 24 h (MOI = 1). The cells were collected, the mRNA levels of **Tnf*α, Il-ib, *Il-6** and *Nlrp3* in cells were detected by qPCR (A-D), and the IL-1β level in the cell supernatant was detected by ELISA (E). ASFV genomic copy numbers were detected by qPCR (F). **(G)** PAMs were infected with ASFV-WT (MOI = 1) or ASFV-intB318L (MOI = 1) for 24 h and then treated with or without LPS for 1 h. The cells were collected to test the expressions of IκB, p65, pB318L, p54, GAPDH, and the phosphorylation of IκB and p65 by Western blotting. Grayscale values were quantified using ImageJ. **(H)** PAMs were infected with ASFV-WT (MOI = 1) or ASFV-intB318L (MOI = 1) for 24 h and then treated with LPS for 6 h. The cells were harvested to extract the cytoplasmic and nuclear fractions and detect the distribution of p65 in the cytoplasm and nucleus. Grayscale values were quantified using ImageJ. Data are representative of three independent experiments with three biological replicates (mean ± s.d.). Ns, not significantly, * *p* < 0.05, *** *p* < 0.001 (one-way ANOVA).

To investigate the role of pB318L in modulating the NF-κB signaling pathway during ASFV infection, PAMs were infected with either ASFV-WT or ASFV-intB318L, an ASFV mutant strain with targeted interruption of *B318L* gene expression that was generated as previously described (S1A Fig) [15]. The results showed that infection with ASFV-intB318L induced significantly higher mRNA levels of *Il-1b*, *Tnf-α*, *Il-6, Nlrp3* compared with infection with ASFV-WT (Fig 2A–2D). Consistent with the above results, ASFV-intB318L infection also resulted in significantly increased secretion of mature IL-1β protein (Fig 2E). Furthermore, there is no significant difference in the gene copy number between ASFV-intB318L and ASFV-WT (Fig 2F). In line with previous findings, the phosphorylation of p65 and IκB induced by LPS was inhibited by ASFV-WT. Although ASFV-intB318L infection also attenuated LPS-triggered p65 and IκB phosphorylation, the inhibitory effect was significantly reduced compared to ASFV-WT (Fig 2G). In addition, ASFV-WT potently inhibits LPS induced nuclear translocation of p65, and impairment of pB318L expression partially restored the suppression of p65 nuclear translocation by ASFV (Fig 2H). Overall, these results demonstrate that pB318L inhibits the activation of NF-κB signaling during ASFV infection.

Due to the fact that ASFV pB318L exhibits significant sequence similarity to geranylgeranyl-diphosphate synthase, implicating potential involvement in protein prenylation modification processes. To investigate the role of the putative enzymatic function of pB318L on the inhibition of NF-κB signaling, we employed three specific inhibitors targeting distinct prenylation modifications process were used: 1) Lovastatin, a competitive inhibitor of HMG-CoA reductase that globally blocks the mevalonate pathway; 2) Lonafarnib, a selective farnesyltransferase inhibitor that specifically impedes protein farnesylation; and 3) GGTI-286, a potent geranylgeranyltransferase I inhibitor that selectively antagonizes geranylgeranylation modification. The results showed that individual administration of these three inhibitors significantly attenuated LPS induced NF-κB promoter activity. Notably, none of these compounds could reverse the dominant inhibitory effect on NF-κB promoter activity exerted by pB318L overexpression (S2A Fig). Based on the conserved active-site residues identified from the homologous structure of pB318L [16], we analyzed the active center of pB318L and predicted that D129, D135, and D212 may determine its enzyme activity. To further elucidate the enzymatic activity of pB318L on NF-κB signaling, three site mutant plasmids (D129A, D135A, D212A) were constructed (S2B Fig). Strikingly, all three mutants (pB318L-D129A, pB318L-D135A, pB318L-D212A) retained the capacity of pB318L to inhibit both NF-κB promoter activation (S2C Fig) and *Il-1b*, *Tnf-α* mRNA expression induced by LPS (S2D and S2E Fig). Collectively, these results indicate that pB318L inhibits NF-κB signaling independently of its putative geranylgeranyl diphosphate synthase activity.

### ASFV pB318L blocks NF-κB activation by targeting NEMO and IKKα

**Fig 3:**
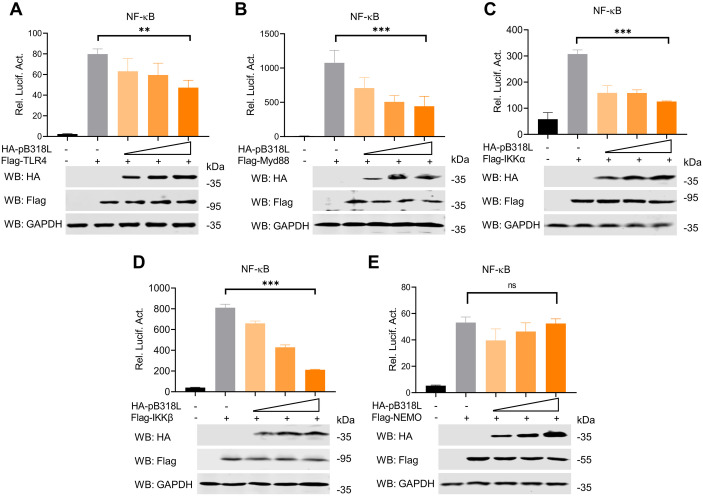
ASFV pB318L inhibits NF-κB promoter activity. **(A-E)** HEK293T cells were transfected with an NF-κB luciferase reporter, a Renilla-TK reporter, a plasmid expressing HA-TLR4 (A), HA-Myd88 (B), HA-IKKα (C), HA-IKKβ (D), or HA-NEMO (E), together with increase amounts (0, 100, 200, and 400 ng) of a plasmid expressing Flag-pB318L. The luciferase activities were detected after 24 h. The expressions of TLR4, Myd88, IKKα, IKKβ, NEMO, pB318L, and GAPDH were detected by Western blotting. Data are representative of three independent experiments with three biological replicates (mean ± s.d.). Ns, not significantly, ** *p* < 0.01, *** *p* < 0.001 (one-way ANOVA).

**Fig 4:**
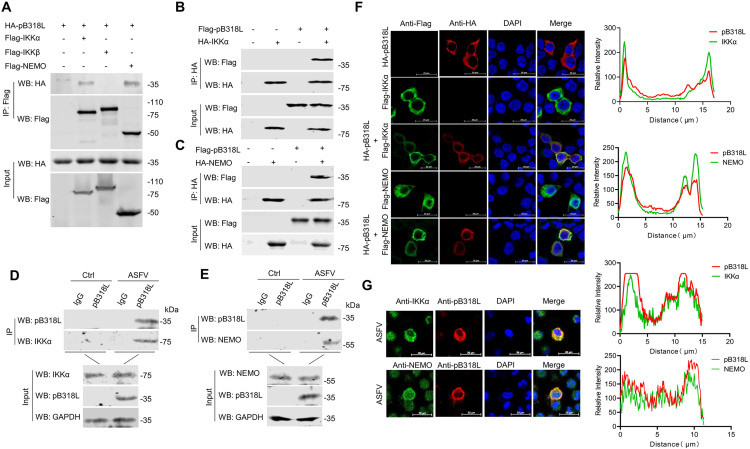
ASFV pB318L interacts with IKKα and NEMO. **(A)** HEK293T cells were transfected with plasmids expressing Flag-pB318L (2 μg) and HA-tagged IKKα, IKKβ or NEMO (2 μg/each) as indicated, respectively. Then, cell lysates were immunoprecipitated with anti-flag (M2) beads and analyzed through Western blotting. **(B-C)** HEK293T cells were transfected with plasmids expressing Flag-pB318L (2 μg) and HA-IKKα, or Flag-pB318L (2 μg) and HA-NEMO (2 μg), respectively. Then, cell lysates were immunoprecipitated with anti-HA (M2) beads and analyzed through Western blotting. **(D-E)** PAMs were infected with ASFV-WT (MOI = 1) for 24 h, cell lysates were immunoprecipitated with anti-pB318L antibody and protein (A + G) Plus-Agarose, then the interaction between ASFV pB318L and endogenous IKKα(D), NEMO(E) in PAMs was analyzed through Western blotting. **(F)** The subcellular localization of HA-pB318L (1 μg) and Flag-IKKα (1 μg) or Flag-NEMO (1 μg) in CRL-2843 cells were detected by immunofluorescence microscopy. Scale bars, 20 μm. The co-localization coefficient was statistically analyzed using ImageJ. **(G)** PAMs were infected with ASFV-WT (MOI = 1), the subcellular localization of pB318L, IKKα and NEMO were detected by immunofluorescence microscopy. Scale bars, 20 μm. The co-localization coefficient was statistically analyzed using ImageJ.

To identify the molecular targets of ASFV pB318L within the NF-κB signaling pathway, HEK293T cells were co-transfected with HA-pB318L and plasmids expressing TLR4, Myd88, IKKα, IKKβ and NEMO. Dual-luciferase reporter assays demonstrated that ectopically expressed pB318L significantly reduced the activation of the NF-κB promoter reporter induced by TLR4, Myd88, IKKα, IKKβ in a dose-dependent manner (Fig 3A–3D) but not by NEMO (Fig 3E). Critically, the western blotting results indicated that ASFV pB318L did not affect the expression levels of these indicated proteins. These findings suggested that ASFV pB318L functions at or upstream NEMO. To confirm direct interactors, HEK293T cells were co-transfected with plasmids expressing pB318L together with IKKα, IKKβ and NEMO, respectively. The results showed that pB318L co-immunoprecipitated with IKKα and NEMO (Fig 4A–4C). To further evaluate the interaction between pB318L and IKKα, NEMO upon ASFV infection, PAMs were infected with ASFV, and the cell lysates were immunoprecipitated with anti-pB318L antibodies. The results showed that ASFV pB318L interacted with endogenous IKKα and NEMO in ASFV-infected PAMs (Fig 4D and 4E). Supporting the findings, the confocal microscopy revealed strong colocalization of HA-pB318L with Flag-IKKα and Flag-NEMO (Fig 4F). Moreover, pB318L co-localized with endogenous IKKα and NEMO in PAMs during ASFV infection (Fig 4G). Finally, the direct interaction between pB318L and IKKα, NEMO was demonstrated through GST pulldown (S3A and S3B Fig). These results suggest that pB318L interacts with IKKα and NEMO to inhibit NF-κB signaling pathway.

### ASFV pB318L disrupts the interaction between IKKα and NEMO

**Fig 5:**
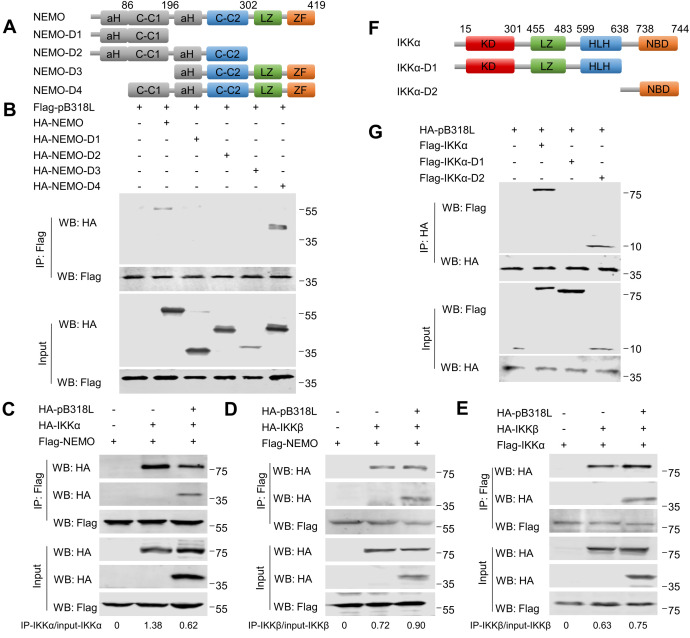
ASFV pB318L inhibits the interaction between IKK α and NEMO. **(A)** Construction of plasmids expressing HA-NEMO and its truncated mutants. **(B)** HEK293T cells were transfected with a plasmid expressing Flag-pB318L (2 μg), along with plasmids expressing HA-NEMO-WT, HA-NEMO-D1, HA-NEMO-D2, HA-NEMO-D3, HA-NEMO-D4 (2 μg/each), respectively. The cells were collected at 24 hpt, and the interactions of NEMO and its deleted mutants with pB318L were analyzed by Co-IP and Western blotting. **(C)** HEK293T cells were transfected with plasmids expressing HA-pB318L, HA-IKKα and Flag-NEMO for 24 h. Then, cell lysates were immunoprecipitated with anti-Flag (M2) beads and analyzed through Western blotting. Grayscale values were quantified using ImageJ. **(D)** HEK293T cells were transfected with plasmids expressing HA-pB318L, HA-IKKβ and Flag-NEMO for 24 h. Then, cell lysates were immunoprecipitated with anti-Flag (M2) beads and analyzed through Western blotting. Grayscale values were quantified using ImageJ. **(E)** HEK293T cells were transfected with plasmids expressing HA-pB318L, HA-IKKβ and Flag-IKKα for 24 h. Then, cell lysates were immunoprecipitated with anti-Flag (M2) beads and analyzed through Western blotting. Grayscale values were quantified using ImageJ. **(F)** Construction of plasmids expressing Flag-IKKα and its truncated mutants. **(G)** HEK293T cells were transfected with a plasmid expressing HA-pB318L (2 μg), along with plasmids expressing Flag-IKKα-WT, Flag-IKKα-D1, Flag-IKKα-D2 (2 μg/each), respectively. The cells were collected at 24 hpt, and the interactions of IKKα and its deleted mutants with pB318L were analyzed by Co-IP and Western blotting.

NEMO consists of four functional domains: two helical domains (CC1 and CC2), a leucine zipper region (LZ), and a zinc finger domain (ZF). In order to map the structural domains required for the interaction between NEMO and pB318L, four NEMO truncated mutant plasmids (D1-D4) were generated (Fig 5A). Co-IP results showed that pB318L interacted with full-length NEMO (WT) and NEMO-D4, which contains C-C1, C-C2, LZ and ZF domains (Fig 5B).

Given the established role of C-C1 and C-C2 domains of NEMO in binding IKKα, IKKβ, we detect whether pB318L disrupts the formation of IKK complex. Co-IP analysis demonstrated that the ectopic expression of pB318L differentially disrupted IKK complex assembly: it inhibited the interaction between IKKα and NEMO, but did not affect the interaction of IKKβ-NEMO and IKKα-IKKβ (Fig 5C–5E).

IKKα includes an amino-terminal kinase domain (KD), a helix-loop-helix (HLH) that is responsible for regulating IKK kinase activity, a leucine zipper (LZ) that mediates kinase dimerization, and a carboxy-terminal NEMO-binding domain (NBD). To detect which domain of IKKα is required for its interaction with pB318L, we constructed a series of domain-truncated plasmids of IKKα. The results of Co-IP showed that, consistent with the results in Fig 5E, pB318L interacts with the NBD domain of IKKα (Fig 5F and 5G). These results further support the conclusion that pB318L interferes with the interaction between IKKα and NEMO.

### ASFV pB318L impairs TRIM21-mediated K63-linked ubiquitination of NEMO

**Fig 6:**
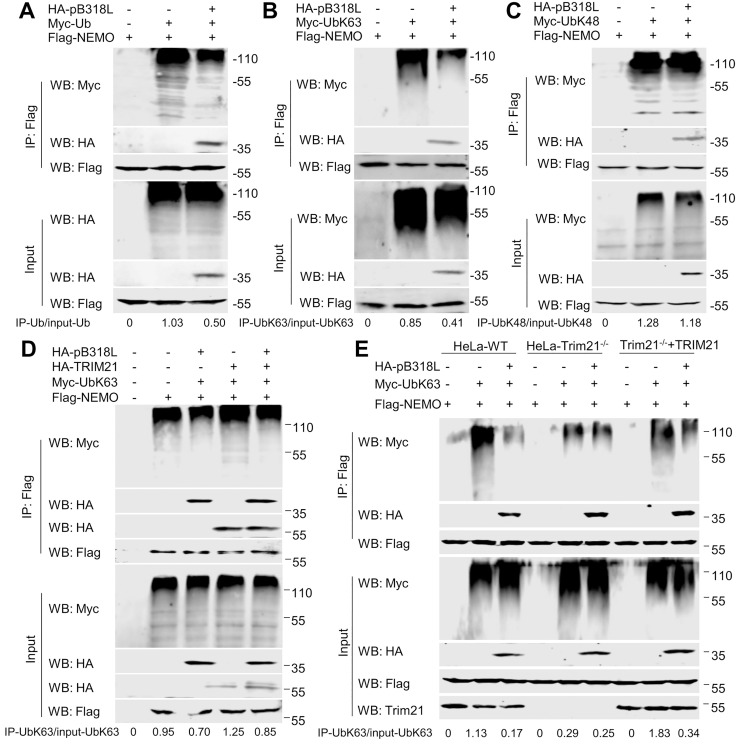
ASFV pB318L inhibits the K63-linked ubiquitination of NEMO. **(A)** HEK293T cells were transfected with plasmids expressing HA-pB318L, Myc-Ub and Flag-NEMO for 24 h. Then, cell lysates were immunoprecipitated with anti-Flag (M2) beads and the ubiquitination of NEMO were analyzed through Western blotting. Grayscale values were quantified using ImageJ. **(B)** HEK293T cells were transfected with plasmids expressing HA-pB318L, Myc-UbK63 and Flag-NEMO for 24 h. Then, cell lysates were immunoprecipitated with anti-Flag (M2) beads and the K63-linked ubiquitination of NEMO were analyzed through Western blotting. Grayscale values were quantified using ImageJ. **(C)** HEK293T cells were transfected with plasmids expressing HA-pB318L, Myc-UbK48 and Flag-NEMO for 24 h. Then, cell lysates were incubated with anti-Flag (M2) beads and the K48 linked ubiquitination of NEMO analyzed through Western blotting. Grayscale values were quantified using ImageJ. **(D)** HEK293T cells were transfected with plasmids expressing HA-pB318L, HA-TRIM21, Myc-UbK63 and Flag-NEMO for 24 h. Then, cell lysates were immunoprecipitated with anti-Flag (M2) beads and the K63-linked ubiquitination of NEMO were analyzed through Western blotting. Grayscale values were quantified using ImageJ. **(E)** Wild-type HeLa cells or HeLa cells deletion of *TRIM21* were transfected with plasmids expressing HA-pB318L, Myc-UbK63 and Flag-NEMO for 24 h. Then, cell lysates were immunoprecipitated with anti-Flag (M2) beads and the K63-linked ubiquitination of NEMO were analyzed through Western blotting. Grayscale values were quantified using ImageJ.

NEMO ubiquitination is critical for canonical NF-κB activation, and the above results identified the LZ and ZF domains as essential for this modification. Therefore, the inhibition of NEMO ubiquitination by pB318L was examined. As expected, the ectopic expression of pB318L inhibits the ubiquitination of NEMO (Fig 6A). In addition, we demonstrated that pB318L suppresses the K63-linked ubiquitination (Fig 6B) but not K48 linked ubiquitination of NEMO (Fig 6C). To screen the protein that is recruited by pB318L to inhibit K63-linked ubiquitination of NEMO, we performed an unbiased proteomic screen through mass spectrometry of anti-pB318L immunoprecipitates. The E3 ubiquitin ligase TRIM21 was identified as a candidate interactor (S4A Fig), and their interaction was verified by Co-IP and GST-pulldown (S4B and S4C Fig).

TRIM21, an E3 ubiquitin ligase, promotes the K63-linked ubiquitination of NEMO upon ectopic expression, while pB318L inhibits the K63-linked ubiquitination of NEMO mediated by TRIM21 (Fig 6D). To confirm the function of TRIM21 on the K63-linked ubiquitination of NEMO, *Trim21* gene knock out HeLa cells were generated. We found that pB318L inhibited the K63-linked ubiquitination of NEMO in wild type HeLa cells, while deletion of *Trim21* gene partially restored the inhibition of pB318L. Upon reintroduced of TRIM21, the inhibitory effect of pB318L on K63-linked ubiquitination of NEMO was restored (Fig 6E). In addition, we detected the protein level of IL-1β in both TRIM21-knockout and TRIM21-rescued cells. The results indicated that knockout of TRIM21 attenuated the inhibitory effect of pB318L on inflammatory cytokine production, while overexpression of TRIM21 enhanced this inhibition (S4D Fig). These findings confirm that TRIM21 acts as a key target E3 ligase of pB318L. Together, these results demonstrate that ASFV pB318L inhibits the K63-linked ubiquitination of NEMO mediated by TRIM21.

### ASFV pB318L suppresses NLRP3 inflammasome activation

**Fig 7:**
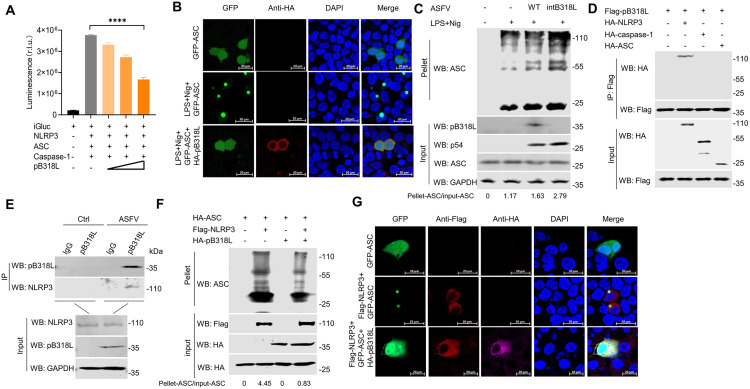
ASFV pB318L inhibits NLRP3 inflammasome activation. **(A)** HEK293T cells were transfected with different doses of a plasmid expressing pB318L in the presence of the components of the iGLuc-based NLRP3 inflammasome system as indicated. At 24 hpt, the supernatants were collected to detect the luciferase activity. **(B)** CRL-2843 cells were transfected with plasmids expressing GFP-ASC and HA-pB318L and stimulated with LPS+nigericin. The oligomerization of ASC was observed by confocal microscopy. **(C)** PAMs were infected with ASFV-WT (MOI = 1) or ASFV-intB318L (MOI = 1) for 24 h and then stimulated with LPS+nigericin, the cell lysates and the pellets were cross-linked using DSS for detecting the oligomerization of ASC by Western blotting. Grayscale values were quantified using ImageJ. **(D)** HEK293T cells were transfected with plasmids expressing Flag-pB318L (2 μg) and HA-tagged NLRP3, ASC or caspase-1 (2 μg/each) as indicated, respectively. Then, cell lysates were immunoprecipitated with anti-flag (M2) beads and analyzed through Western blotting. **(E)** PAMs were infected with ASFV-WT (MOI = 1) for 24 h, cell lysates were immunoprecipitated with anti-pB318L antibody and protein (A + G) Plus-Agarose, then the interaction between ASFV pB318L and endogenous NLRP3 in PAMs was analyzed through Western blotting. **(F)** HEK293T cells were transfected with plasmids encoding HA-ASC, Flag-NLRP3 and HA-pB318L as indicated. The cell lysates and the pellets were cross-linked using fresh dextran sulfate sodium (DSS) for detecting the oligomerization of ASC by Western blotting. Grayscale values were quantified using ImageJ. **(G)** CRL-2843 cells were transfected with plasmids expressing GFP-ASC, Flag-NLRP3 and HA-pB318L. The oligomerization of ASC was observed by confocal microscopy. Data are representative of three independent experiments with three biological replicates (mean ± s.d.). **** *p* < 0.0001 (one-way ANOVA).

Our previous research has shown that ASFV infection activates NLRP3 inflammasome and induces the maturation and release of IL-1β. To detect the effect of pB318L on the activation of NLRP3 inflammasome, HEK293T cells were co-transfected with plasmids expressing the core components of NLRP3 inflammasome, including NLRP3, ASC, caspase-1, pro-IL-1β-gaussia luciferase (iGluc) reporter along with a plasmid expressing pB318L. Subsequent measurement of luciferase activity revealed that pB318L significantly inhibited the iGluc reporter activity induced by NLRP3 inflammasome activation (Fig 7A), indicating that pB318L inhibited NLRP3 inflammasome activity. To determine whether the enzymatic activity of pB318L is required for its inhibition of NLRP3 inflammasome, we assessed the effect of prenylation inhibitors. The results showed that none of the three prenylation inhibitors could rescue the inhibition of iGluc activity inhibited by pB318L (S5A Fig). Furthermore, consistent with pB318L-WT, all of the three enzyme-activity-deficient mutants of pB318L also inhibited iGluc activity induced by NLRP3 inflammasome activation (S5B Fig). These findings collectively indicate that the enzymatic activity of pB318L is dispensable for its inhibition of the NLRP3 inflammasome. To investigate the regulatory effect of pB318L on ASC oligomerization, CRL-2843 cells were co-transfected with the plasmids expressing GFP-ASC and HA-pB318L, and then stimulated with LPS and nigericin. Confocal microscopy analysis revealed that LPS+nigericin treatment induced the formation of characteristic ASC specks in control cells. However, co-expression of pB318L significantly suppressed ASC oligomerization, maintaining the adaptor protein in a diffuse cytoplasmic distribution (Fig 7B). To confirm this effect in the context of viral infection, PAMs were infected with ASFV-WT or ASFV-intB318L, and subsequently treated with LPS and nigericin. The results revealed that the ASC oligomerization levels in PAMs infected with ASFV-intB318L were significantly higher compared to those infected with ASFV-WT (Fig 7C). Taken together, these results demonstrate that pB318L inhibited ASC oligomerization, and this inhibitory function is independent of its enzymatic activity.

To identify which component of the NLRP3 inflammasome interacts with pB318L, HEK293T cells were co-transfected with pB318L and NLRP3, ASC, or caspase-1 for Co-IP assay. As shown in Fig 7D, overexpressed pB318L specifically co-immunoprecipitated with NLRP3, but not with ASC or caspase-1. Consistent with this result, pB318L also co-immunoprecipitated with endogenous NLRP3 in PAMs infected with ASFV (Fig 7E). Furthermore, we demonstrated that pB318L effectively inhibits NLRP3-induced ASC oligomerization in both HEK293T and CRL-2843 cells through disuccinimidyl suberate (DSS) crosslinking and confocal imaging (Fig 7F and 7G). Collectively, these results demonstrate that pB318L directly interacts with NLRP3 to inhibit the activation of NLRP3 inflammasomes.

### ASFV pB318L specifically inhibits NLRP3 oligomerization

**Fig 8:**
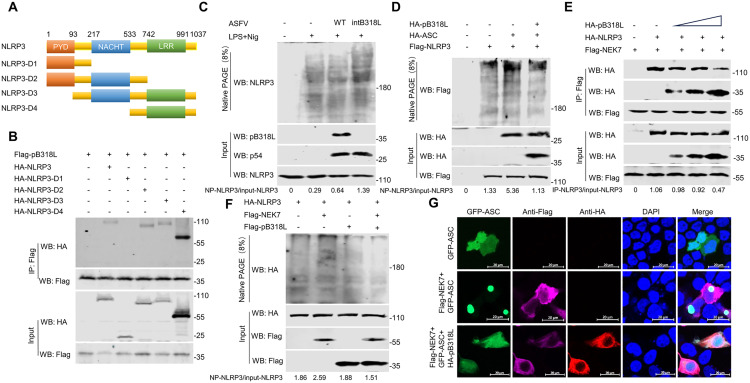
ASFV pB318L inhibits NLRP3 oligomerization. **(A)** Construction of plasmids expressing HA-NLRP3 and its truncated mutants. **(B)** HEK293T cells were transfected with a plasmid expressing Flag-pB318L (2 μg), along with plasmids expressing HA-NLRP3-WT, HA-NLRP3-D1, HA-NLRP3-D2, HA-NLRP3-D3, HA-NLRP3-D4 (2 μg/each), respectively. The cells were collected at 24 hpt, and the interactions of NLRP3 and its deleted mutants with pB318L were analyzed by Co-IP and Western blotting. **(C)** PAMs were infected with ASFV-WT or ASFV-intB318L for 24 h, and then the cells were collected to detect the oligomerization of NLRP3 by Native PAGE. Grayscale values were quantified using ImageJ. **(D)** HEK293T cells were transfected with plasmids encoding HA-ASC, Flag-NLRP3 and HA-pB318L as indicated. The cells were lysed to detect the oligomerization of NLRP3 by Native PAGE. Grayscale values were quantified using ImageJ. **(E)** HEK293T cells were transfected with plasmids expressing HA-pB318L, HA-NLRP3 and Flag-NEK7. Then, cell lysates were immunoprecipitated with anti-flag (M2) beads and analyzed through Western blotting. Grayscale values were quantified using ImageJ. **(F)** HEK293T cells were transfected with plasmids expressing Flag-pB318L, HA-NLRP3 and Flag-NEK7. The cells were lysed to detect the oligomerization of NLRP3 by Native PAGE. Grayscale values were quantified using ImageJ. **(G)** CRL-2843 cells were transfected with plasmids expressing GFP-ASC, Flag-NEK7 and HA-pB318L. The oligomerization of ASC was observed by confocal microscopy.

NLRP3 contains three functional domains: the nucleotide binding domain (NACHT), the leucine rich repeat domain (LRR), and the pyridine domain (PYD). In order to map the NLRP3 domain required for its interaction with pB318L, four NLRP3 truncation mutants were constructed (Fig 8A) and systematic Co-IP assays were performed. The results revealed that pB318L specifically interacts with the NACHT and LRR domains of NLRP3, but not with the PYD domain (Fig 8B). Given the essential roles of the NACHT and LRR domains in the oligomerization of NLRP3, we next investigated the effect of pB318L on this process. The Native-PAGE analysis demonstrated enhanced NLRP3 oligomerization in ASFV-intB318L-infected cells compared to that in ASFV-WT-infected cells (Fig 8C). Conversely, overexpression of pB318L in HEK293T inhibits NLRP3 oligomerization (Fig 8D). NEK7 is a member of the mammalian NIMA associated kinase (NEK protein) family, which regulates NLRP3 oligomerization and activation by binding to the NACHT and LRR domains of NLRP3. We hypothesized competitive inhibition between pB318L and NEK7. As expected, the ectopic expression of pB318L dose-dependently disrupted the interaction between NEK7 and NLRP3 (Fig 8E). Consequently, this competitive binding effectively abrogated NEK7-mediated NLRP3 oligomerization (Fig 8F). To functionally validate the effect of pB318L, the confocal imaging was employed. The results showed that overexpression of NEK7 significantly enhanced ASC speck formation, while co-expression of pB318L abolished ASC specks induced by NEK7, leading to its diffuse cytosolic distribution (Figs 8G and S6). In summary, the above results demonstrate that ASFV pB318L inhibits the interaction between NEK7 and NLRP3, resulting in a decrease in NLRP3 oligomerization and thus suppressing the activation of NLRP3 inflammasomes.

## Discussion

African swine fever causes highly contagious and widespread hemorrhagic viral disease in both wild and domestic pigs, with a mortality rate that can reach up to 100%. This disease poses a devastating threat to the global pig industry [2]. Currently, ASF is spreading in China and other Asian countries, highlighting the urgent need for the development of effective treatment and prevention strategies [17]. ASFV can replicate within immune cells, particularly macrophages, without being recognized by the host’s immune system, demonstrating that ASFV has evolved a strategy to escape the host’s innate immune response. It is reported that the ability of ASFV regulating innate immunity is closely related to its pathogenicity [18]. Therefore, targeted deletion of specific genes involved in immune evasion represents a rational strategy for developing attenuated vaccines candidates with enhanced immunogenicity and protective efficacy.

Following viral invasion, the host’s immune system initiates antiviral innate immunity and inflammatory response by detecting pathogen-associated molecular patterns (PAMPs) through pattern recognition receptors (PRRs) [19]. The activation of PRRs triggers downstream signaling cascades, ultimately leading to the expression of IFNs and pro-inflammatory cytokines [20]. These antiviral effectors play a crucial role in coordinating effective innate and adaptive immune responses to combat viral infections. Understanding the complex interactions between virus and host is vital for the development of innovative vaccines and antiviral therapies [21]. Removing immunosuppressive genes from ASFV to develop low-toxicity variants is undoubtedly a promising strategy for producing ASF vaccines. This approach is exemplified by the recombinant virus OUR T88/3 ΔDP2 with DP71L (NL) and DP96R (UK) genes deletion, which provides 66% heterologous protection and 100% homologous protection [22]. The DP71L and DP96R genes are critical virulence genes [23,24] and the related encoding proteins have also been identified as immune-suppressive proteins. The protein pDP71L can directly interact with eIF2α, recruit PP1α to dephosphorylate eIF2α, and inhibit the eIF2α-ATF4-CHOP cell apoptosis pathway [25]. Meanwhile, pDP96R has been reported to inhibit cGAS-STING-mediated IFN-I production and NF-κB signaling by blocking TBK1 and IKKβ activation [26]. Similarly, pMGF505-7R (pA528R) reduces the production of IL-1β through interacting with the IKK complex and NLRP3, and inhibits the production of IFN-Ⅰ by blocking the nuclear translocation of IRF3 [27] and degrading STING expression [28]. The protein pH240R attenuates host immune response by suppressing NF-κB signaling transduction, NLRP3 inflammasome activation [29], and IFN-I/ISGs production [30,31]. Notably, the double gene-deletion mutant ASFV-ΔH240R-Δ7R derived from the highly virulent HLJ/18 strain demonstrated complete protection (10^5^ HAD_50_) with no adverse effects in piglets [32]. In our previous studies, we identified the immunosuppressive role of pB318L. It interacted with STING to inhibit the translocation of STING from the endoplasmic reticulum to the Golgi apparatus, as well as interacted with IFNAR1/2 to disrupt the interaction of IFNAR1-TYK2 and IFNAR2-JAK1, resulting in decreased production of IFN-I and ISGs [15]. ASFV encodes a large number of proteins that inhibit host innate immunity. These proteins may collectively suppress the host’s innate immune response during ASFV infection to facilitate viral replication. Deletion of a single viral immune evasion gene typically results in attenuated viral virulence; however, residual virulence often persists, which is attributed to the functional compensation of other ASFV-encoded immune antagonists. Consequently, the development of multi-gene deletion ASFV strains designed to eliminate overlapping immune evasion activities has emerged as a prevailing strategy for generating promising vaccine candidates. We have incorporated these considerations and relevant discussions into the Discussion section of the revised manuscript. The current study reveals that pB318L binds NEMO and NLRP3 endows it with the ability to inhibit inflammatory responses. Previous animal experiments indicated that ASFV-intB318L exhibited reduced toxicity in piglets, achieving a survival rate of 60% [15]. These findings position B318L as a high-priority target for rational vaccine design. Its deletion, particularly in combination with other virulence genes, represents a viable strategy for developing safe and efficacious candidate ASFV vaccine.

ASFV infection induces the release of various pro-inflammatory cytokines, including TNF-α, IL-1β, IL-6, and IL-8. TNF-α and IL-1β are pivotal components of the host’s innate antiviral immune response and are crucial for regulating the replication of diverse viruses, such as classical swine fever virus [33] and Japanese encephalitis virus [34]. While ASFV is capable of inducing potent inflammatory responses in pigs, the mechanisms by which it regulates inflammation are complex. An increasing number of studies indicated that ASFV encodes multiple proteins that can effectively inhibit inflammatory responses [27]. For instance, ASFV pF317L has been shown to interact with IKKβ and inhibits its activation. This interaction subsequently impairs the stability of IκBα and the activation of NF-κB, thereby reducing the expression of various pro-inflammatory cytokines and promoting viral replication [35]. Another notable protein is ASFV pS273R, which has been identified as a cysteine protease belonging to the SUMO-1 specific protease family. ASFV pS273R specifically cleaves GSDMD in an enzymatic activity-dependent manner, thereby inhibiting cell pyroptosis and regulating the host’s inflammatory response [36]. ASFV pH240R also plays a significant role in this context. It interacts with NEMO, resulting in reduced phosphorylation of IκBα and p65. In addition, pH240R binds to NLRP3, thereby inhibiting NLRP3 inflammasome activation and subsequently reducing IL-1β production [29]. ASFV *A238L* gene encodes a protein that is homologous to the NF-κB inhibitor IκB. pA238L interacts with NF-κB to prevent its transcription and downregulate the production of pro-inflammatory cytokines [37]. In this study, we demonstrate that ASFV pB318L targets key components of both NF-κB and inflammasome pathways. It interacts with NEMO, inhibits the interaction between IKKα and NEMO, and suppresses the K63-linked ubiquitination of NEMO. In addition, pB318L interacts with the NACHT and LRR domains of NLRP3, inhibiting the oligomerization of NLRP3 by suppressing the interaction between NEK7 and NLRP3.

Prenylation is a critical lipid post-translational modification of many membrane proteins. Prenyl transferases catalyze the addition of isoprene groups (farnesyl or geranylgeranyl) to protein substrates, conferring hydrophobicity essential for targeting proteins to cellular or organellar membranes. This modification plays a pivotal role in various cellular processes, particularly in key cell signaling pathways for cancer development and microbial infection activation [38]. Furthermore, prenylation modification has also been shown to regulate the process of inflammation. Skinner *et al*. demonstrated that the loss of prenylation in some GTPases such as Rac1 or RhoA leads to the activation of inflammasomes, resulting in caspase-1 processing and increased IL-1β production [39]. Other studies suggest that isoprenoids deficiency enhances RhoA activity, leading to further increase in gene expression of pro-IL-1β and activation of Rac1, thereby inducing pro-caspase-1 activation within the inflammasomes [40]. Contrasting with these findings, our results show that the use of isopentenyl inhibitors has a negative regulatory effect on inflammatory response. This discrepancy may be attributed to the differences in the experimental cell models employed. The HEK293T cells we used in this study lack a fully complete inflammatory system. In addition, potential deficiencies in the mevalonate or isopentenylation pathways in HEK293T cells could also contribute to the different outcomes. In the context of ASFV infection, the *B318L* gene encodes a protein that is homologous to GGPPS, which can synthesize GGPP. Our findings reveal that overexpress of the enzyme activity mutant plasmid of pB318L, retains the same inhibitory effect on the inflammatory response as that of pB318L-WT, indicating that the ability of pB318L to inhibit inflammatory response is independent of its enzymatic activity.

It has been reported that TRIM21 can bind to antibodies, leading to its B-box domain phosphorylation, which releases its self-inhibition and subsequently triggers its self-ubiquitination by the E2 enzymes Ube2W and Ube2N/Ube2V2. This ubiquitination directs the TRIM21-antibody complex to the Valosin-Containing Protein (VCP) and proteasome for degradation. Subsequently, Poh1 removes the K63-linked ubiquitin chain from TRIM21 to activate NEMO [41–43]. Given that TRIM21 specifically binds to the Fc fragment of antibodies, we sought to eliminate the potential impact of this function on protein interactions. To this end, GST pull-down was employed to assess the direct interaction between pB318L and TRIM21. The results indicated that pB318L directly interacts with TRIM21 and inhibits TRIM21-mediated K63-linked ubiquitination of NEMO.

**Fig 9:**
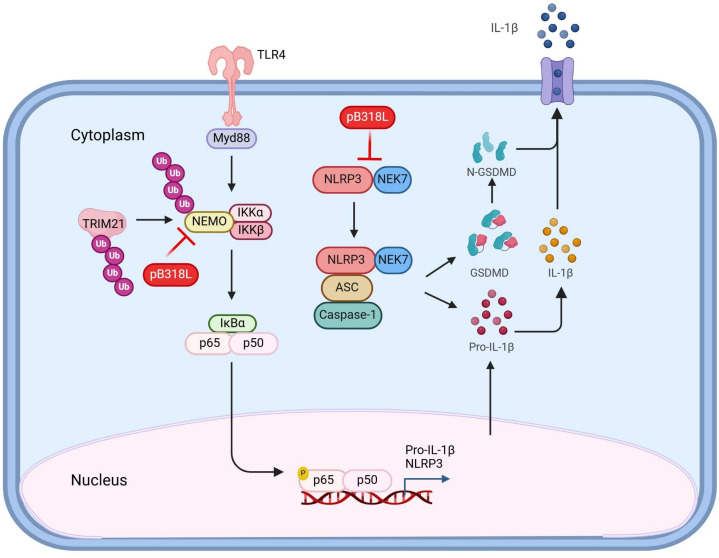
Molecular mechanism of pB318L-mediated inhibition of inflammatory response. ASFV pB318L exerts a negative regulatory effect on the inflammatory response through multiple mechanisms. On one hand, pB318L directly interacts with NEMO, thereby inhibiting the formation of IKKα-NEMO complex and TRIM21-mediated K63-linked ubiquitination of NEMO, which prevents the activation of NF-κB signaling pathways. On the other hand, pB318L binds to the NACHT and LRR domains of NLRP3, disrupting the interaction between NEK7 and NLRP3. This disruption inhibits the oligomerization of NLRP3, thereby suppressing the activation of the NLRP3 inflammasome and the subsequent production of pro-inflammatory cytokines. This illustration was Created in BioRender. Weng, C. (2025) https://BioRender.com/uc76y2m.

In summary, our study provides compelling evidence that ASFV pB318L employs multiple strategies to inhibit inflammatory responses. Specifically, pB318L suppresses the NEMO-IKKα complex assembly and K63-linked ubiquitination of NEMO through its direct interaction with NEMO. In addition, pB318L binds to NLRP3 and disrupts the interaction between NEK7 and NLRP3, thereby inhibiting the activation of NLRP3 inflammasome (Fig 9). Importantly, our data indicate that the inhibition of inflammatory response by pB318L is independent of its GGPPS enzyme activity. Consistent with this, the ASFV mutant strain with an interrupted *B318L* gene reading frame induced significantly higher levels of pro-inflammatory cytokines compared to wild-type virus. Collectively, our findings reveal a novel mechanism of ASFV pB318L evading key host inflammatory defenses by targeting both NF-κB activation and NLRP3 inflammasome assembly, which highlighting its multifaceted role in modulating host immune signaling pathways.

## Materials and methods

### Ethics statements

All experiments involving ASFV HLJ/18 (Genbank accession number: MK333180.1) and ASFV-intB318L were performed under the enhanced biosafety level 3 (P3+) and level 4 (P4) containment at the Harbin Veterinary Research Institute (HVRI), Chinese Academy of Agricultural Sciences (CAAS). This work was conducted in compliance with institutional biosafety protocols approved by the Ministry of Agriculture and Rural Affairs.

### Reagents and antibodies

Dulbecco’s Modified Eagle’s Medium (DMEM) (C11995500CP), RPMI 1640 (C11875500CP), and fetal bovine serum (FBS) (10091-148) were purchased from GIBCO (Grand Island, NE, USA). Anti-Flag (M2) beads (M8823) were purchased from Sigma-Aldrich (St. Louis, MO, USA). Lovastatin (S2061) and Lonafarnib (S2797) were purchased from Selleckchem (Houston, TX, USA). Protease inhibitor Cocktail (4693132001) was purchased from Roche (Basel, Switzerland). Dual-Luciferase Reporter Assay System (E1910) was purchased from Promega (Madison, MI, USA). PrimeScript RT Reagent Kit (RR037A) and SYBR Premix Ex Taq II (RR820A) were purchased from Takara (Shiga, Japan). The following antibodies were purchased from Sigma-Aldrich (St. Louis, MO, USA): rabbit anti-Flag (F7425-2MG), mouse anti-Flag (F1804-1MG), rabbit anti-HA (SAB4300603), mouse anti-HA (HS658-2ML). Mouse anti-GAPDH (60004-1-Ig) was purchased from Proteintech (Wuhan, China). The following antibodies were purchased from Cell Signaling Technology (Danvers, MA, USA): rabbit anti-p65 (D14E12), rabbit anti-phospho-NF-κB (Ser536) (93H1), rabbit anti-IκBα (44D4), rabbit anti-phospho-IκBα (Ser32) (14D4), rabbit anti-IKKα (D3W6N), rabbit anti-IKKβ (D30C6). Anti-pB318L, anti-p54, and anti-pig NLRP3 antibodies are all produced and stored in our laboratory. The IRDye 800CW goat anti-rabbit IgG (H+L) (925-32211) and IRDye 800CW goat anti-mouse IgG (H+L) (925-32210) were purchased from LI-COR (Lincoln, NE, USA). Alexa Flour 488 goat anti-Rabbit IgG(H+L) (A11008) and Alexa Flour 594 goat anti-Mouse IgG(H+L) (A11032) were purchased from Thermo Fisher Scientific (Waltham, MA, USA). Porcine IL-1β ELISA Kit (ELP-IL1b) was purchased from RayBio (Norcross, GA, USA). Nuclear and Cytoplasmic Extraction Reagents (VL315819) were purchased from Thermo Fisher Scientific (Waltham, MA, USA).

### Plasmids

The NF-κB luciferase reporter plasmids, and TK-Renilla luciferase reporter plasmid were generously provided by Professor Hong Tang. To construct plasmids expressing Flag-tagged or hemagglutinin (HA)-tagged proteins involved in the NF-κB and NLRP3 signaling pathways, the corresponding swine cDNAs were amplified by standard reverse transcription-PCR (RT-PCR) using total RNA extracted from PAMs as templates. The amplified cDNAs were then cloned into the pCAGGS-Flag or pCAGGS-HA vector, respectively. To generate plasmids expressing Flag-tagged pB318L and HA-tagged pB318L, the cDNAs of these genes were amplified and cloned into pCAGGS-Flag vector or pCAGGS-HA vector, respectively. To express Flag-tagged pB318L mutants, we designed primers targeting specific nucleotide sequences, including Asp129, Asp135, and Asp212. By using site-directed mutagenesis, the nucleotides encoding aspartic acid (Asp) were mutated to those encoding alanine (Ala). The mutated fragments were amplified and cloned into the pCAGGS-Flag vector. All constructs were validated by DNA sequencing. The primers used in this study are listed in S1 Table.

### Cell lines and viruses

HEK293T cells and CRL-2843 cells were purchased from American type culture collection (ATCC, Rockville, MD, USA) and cultured in DMEM. Primary pulmonary alveolar macrophages (PAMs) isolated from specific pathogen-free (SPF) piglets (without ASFV, PRRSV, PRV, PCV2, and other 28 pathogens) were cultured in RPMI 1640 supplemented with 10% FBS, 100 U/mL penicillin, and 100 μg/mL streptomycin at 37°C with 5% CO_2_. ASFV HLJ/18 (GenBank accession number: MK333180.1) strain was isolated from a pig sample from an ASF outbreak farm in China [44].

### Co-immunoprecipitation (Co-IP) and immunoblot analysis

For Co-IP, the cells were collected and lysed in lysis buffer (50 mM Tris-HCl, pH 7.4, 150 mM NaCl, 5 mM MgCl_2_, 1 mM EDTA, 1% Tris-HCl, and 10% glycerol) containing 1 mM PMSF and 1 × protease inhibitor cocktail (Basel, Switzerland, Roche). Then, cell lysates were incubated with anti-flag (M2) beads (Sigma-Aldrich, St. Louis, MO, USA) or added indicated antibodies and protein A + G Plus-Agarose (Santa Cruz Biotechnology, Dallas, TX, USA). After incubation 4–8 h at 4°C, the beads were washed three times with lysis buffer. For immunoblot analysis, the samples were separated by 10–12% sodium sulfate polyacrylamide gel electrophoresis (SDS-PAGE) and then transferred to a polyvinyl difluoride (PVDF) membrane (Sigma-Aldrich, St. Louis, MO, USA). After incubation with primary and secondary antibodies, the membrane is visualized by the Odyssey Dual Color Infrared Fluorescence Imaging System (LI-COR, Lincoln, NE, USA).

### Confocal microscopy and co-localization analysis

The cells were transfected with indicated plasmids, and the cell supernatant was discarded at 24 hpt. The cells were fixed for 10 min in 4% paraformaldehyde and then permeabilized for 15 min with 0.3% Triton X-100. After blocking in 1 × PBS with 10% FBS for 30 min, the cells were incubated with the appropriate primary antibodies and then stained with secondary antibodies as indicated. The subcellular co-localization was visualized using a Zeiss LSM-880 laser scanning fluorescence microscope (Carl Zeiss AG, Oberkochen, Germany) under a 63 × oil objective.

### GST pull-down Assay

The ASFV pB318L transmembrane region-removed sequence was cloned into the pGEX-6p-1 vector and transformed into BL21 competent cells. The obtained bacteria were expanded, cultured, and induced with 0.5 mM IPTG for 20 h. After centrifugation, the cells were resuspended in PBS and then sonicated to obtain bacterial lysates containing GST-pB318L. Flag-tagged IKKα, NEMO and TRIM21 was transfected into HEK293T cells, and cells were lysed in lysis buffer (50 mM Tris-HCl, pH 7.4, 150 mM NaCl, 5 mM MgCl_2_, 1 mM EDTA, 1% Tris-HCl and 10% glycerol) containing 1 mM PMSF and 1 × protease inhibitor cocktail (Basel, Switzerland, Roche) at 24 hpt. The cell lysates were mixed with bacterial lysates containing GST-pB318L and added to GST beads (GenScript, NJ, USA) to incubate for 6–8 h at 4°C. The beads were washed five times with lysis buffer and then subjected to Western blotting analysis.

### Luciferase reporter gene assay

HEK293T cells were co-transfected with the indicated plasmids. After 24 h, the cells were lysed in lysis buffer, and luciferase activities of NF-κB-luciferase-reporter (100 ng), and TK-Renilla reporter (5 ng) were measured with a Dual-Luciferase Reporter Assay System (Promega, Madison, MI, USA) according to the manufacturer’s instructions. The data were normalized to the transfection efficiency by dividing the firefly luciferase activity by the Renilla luciferase activity. Each experiment was conducted three times independently, and the representative results were shown.

### ASC speck and oligomerization

CRL-2843 cells were transfected with plasmids encoding GFP-ASC and pB318L. At 24 hpt, the cells were stimulated with LPS (100ng/ml) for 8 h and nigericin (5 μM) for an extra 4 h before harvest. ASC was visualized using a Zeiss LSM-880 laser scanning fluorescence microscope. HEK293T was transfected with plasmids expressing NLRP3, pB318L, and ASC. After 24 h, cells were collected and lysed with NP-40 free lysis buffer. The pellets were washed with PBS for three times and cross-linked using fresh DSS (2 mM, Sigma) at 37°C for 30 min. Then the cross-linked pellets were centrifuged and mixed with SDS-loading buffer for western blot analysis.

### Native PAGE detection of NLRP3 oligomerization

HEK293T cells were transfected with plasmids expressing NLRP3, ASC, and pB318L, or plasmids expressing NLRP3, NEK7, and pB318L. Cells were collected 24 h later. PAMs were infected with either ASFV-WT or ASFV-intB318L, followed by stimulation with LPS and nigericin. Following cell lysis, centrifugation was performed, and native loading buffer was added to the resulting supernatant. SDS-free gels were used for electrophoresis, and neither the electrophoresis buffer nor the transfer buffer contained SDS.

### RNA extraction and qPCR

Total RNA was extracted using TRIzol reagent (Thermo Fisher Scientific, Waltham, MA, USA), and reverse transcription was accomplished with the PrimeScript RT Reagent Kit (Takara, Shiga, Japan). The reverse transcription products were amplified using the Agilent-Strata gene Mx Real-Time qPCR system with SYBR Premix Ex Taq Ⅱ (Takara, Shiga, Japan) according to the manufacturer’s instructions. The data were normalized according to the level of β-actin expression in each individual sample. All experiments were performed at least in triplicate. All the qPCR primers are listed in S2 Table.

### ELISA

The concentrations of IL-1β (Ray Biotech, Norcross, GA) in the serum were measured by ELISA kits according to the manufacturer’s instructions.

### Nuclear and cytoplasmic extraction

HeLa cells were transfected with the indicated plasmids for 24 h and then stimulated with LPS for another 6 h. After that, the cells were harvested and processed using Nuclear and Cytoplasmic Extraction Reagent (Thermo Scientific, Waltham, MA, USA). PAMs were infected with ASFV-WT or ASFV-intB318L for 24 h (MOI = 1) and then stimulated with LPS for another 6 h, and processed using Nuclear and Cytoplasmic Extraction Reagent. Nuclear and cytoplasmic extracts were examined by Western blotting.

### Statistical analysis

Statistical analysis was conducted using the unpaired Student’s t-test and one-way analysis of variance (ANOVA) followed by the Bonferroni post-test. P values less than 0.05 were considered statistically significant. Sample sizes were chosen by standard methods to ensure adequate power, and no exclusion, randomization of weight, sex, or blinding was used for the animal studies.

## Supporting information

S1 Fig Schematic representation of the generation of ASFV-intB318L virus. A recombinant African swine fever virus (ASFV) was engineered via homologous recombination in PAMs. The plasmid pBluescript II KS (+) served as the backbone, into which a cassette containing the overlapping sequence of B318L and B438L genes, two LoxP sites, and the enhanced EGFP gene under the control of the ASFV p72 promoter was inserted upstream of the B318L locus. Additionally, the first nucleotide “A” and the tenth nucleotide “C” of the B318L open reading frame (ORF) were deleted to disrupt its coding sequence. Recombinant transfer vector (pB-intB318L-eGFP) containing about 800 bp left homologous arm at the left of 96328 site (A) of ASFV HLJ/18 genome, a reporter gene cassette, followed by about 800 bp right homologous arm at the right of 96239 site (C) of ASFV HLJ/18 genome.
(TIF)

S2 Fig The enzyme activity of pB318L does not affect its inhibition of the NF-κB signaling pathway. **(A)** HEK293T cells were transfected with an NF-κB luciferase reporter, a Renilla-TK reporter, and plasmid expressing Flag-pB318L. After 24 h, the cells were treated with LPS for 6 h, and then were treated with Lovastatin, Lonfarnib, GGTI-286 for another 12 h, the luciferase activities were detected. **(B)** The expression of HA-pB318L enzyme activity mutant plasmid. **(C)** HEK293T cells were transfected with an NF-κB luciferase reporter, a Renilla-TK reporter, and a plasmid expressing Flag-pB318L-WT or Flag-pB318L-Mut and were treated with LPS for 6 h, the luciferase activities were detected after 24 h. **(D-E)** HeLa cells were transfected with plasmids expressing Flag-pB318L-WT or Flag-pB318L-Mut, and then treated with LPS for 6 h, the mRNA levels of *Il-1b* and *Tnfα* were analyzed by qPCR.
(TIF)

S3 Fig ASFV pB318L directly interacts with IKKα and NEMO. **(A)** HEK293T cells were transfected with plasmids expressing Flag-IKKα for 24h, Then, cell lysates were immunoprecipitated with GST or GST-pB318L protein and anti-GST beads. Direct interaction between IKKα and ASFV pB318L was detected by GST pull-down assay. **(B)** HEK293T cells were transfected with plasmids expressing Flag-NEMO for 24h, Then, cell lysates were immunoprecipitated with GST or GST-pB318L protein and anti-GST beads. Direct interaction between NEMO and ASFV pB318L was detected by GST pull-down assay.
(TIF)

S4 Fig ASFV pB318L recruits TRIM21. **(A)** PAMs were infected with ASFV-WT and collected to binding with anti-pB318L antibody, mass spectrometry analysis was performed after SDS-PAGE. TRIM21 was found to interact with pB318L. **(B)** HEK293T cells were transfected with plasmids expressing HA-pB318L and Flag-TRIM21 for 24 h. Then, cell lysates were incubated with anti-Flag (M2) beads and analyzed through Western blotting. **(C)** HEK293T cells were transfected with plasmids expressing Flag-TRIM21 for 24h, Then, cell lysates were immunoprecipitated with GST or GST-pB318L protein and anti-GST beads. Direct interaction between TRIM21 and ASFV pB318L was detected by GST pull-down assay. (D) HeLa cells, HeLa deletion of *TRIM21* or TRIM21 rescue in HeLa-TRIM21 cells were transfected with plasmids expressing HA-pB318L and treated witn LPS + ATP. Cell supernatants were collected and the protein levels of IL-1β in the supernatants was detected.
(TIF)

S5 Fig The enzyme activity of pB318L does not affect its inhibition of NLRP3 inflammasome. **(A)** HEK293T cells were transfected with iGLuc-based NLRP3 inflammasome system, and plasmid expressing Flag-pB318L. After 24 h, the cells were treated with Lovastatin, Lonfarnib, GGTI-286 for 12 h, the luciferase activities were detected. **(B)** HEK293T cells were transfected with iGLuc-based NLRP3 inflammasome system, and plasmids expressing Flag-pB318L-WT or Flag-pB318L-Mut and the luciferase activities were detected after 24 h.
(TIF)

S6 Fig ASFV pB318L inhibits NEK7-induced ASC oligomerization. **(A)** CRL-2843 cells were transfected with plasmids expressing GFP-ASC, Flag-NEK7 and HA-pB318L. The oligomerization of ASC was observed by confocal microscopy. **(B)** Randomly select three large fields of view, and quantify the percentage of NEK7-induced ASC specks as well as that of NEK7-induced ASC specks under the condition of pB318L co-expression.
(TIF)

S1 Table Primers used for plasmid construction in this study. (DOCX)

S2 Table Primers used for qPCR in this study. (DOCX)

S1 Data Source data. (XLSX)
