# Supplementary figures and images for: African swine fever virus pB318L suppresses inflammatory response by inhibiting NF-κB activation and NLRP3 inflammasome formation
Source: PLoS Pathog. 2025 Oct 22;21(10):e1013558. doi: 10.1371/journal.ppat.1013558 (PMC12543117; doi:10.1371/journal.ppat.1013558)

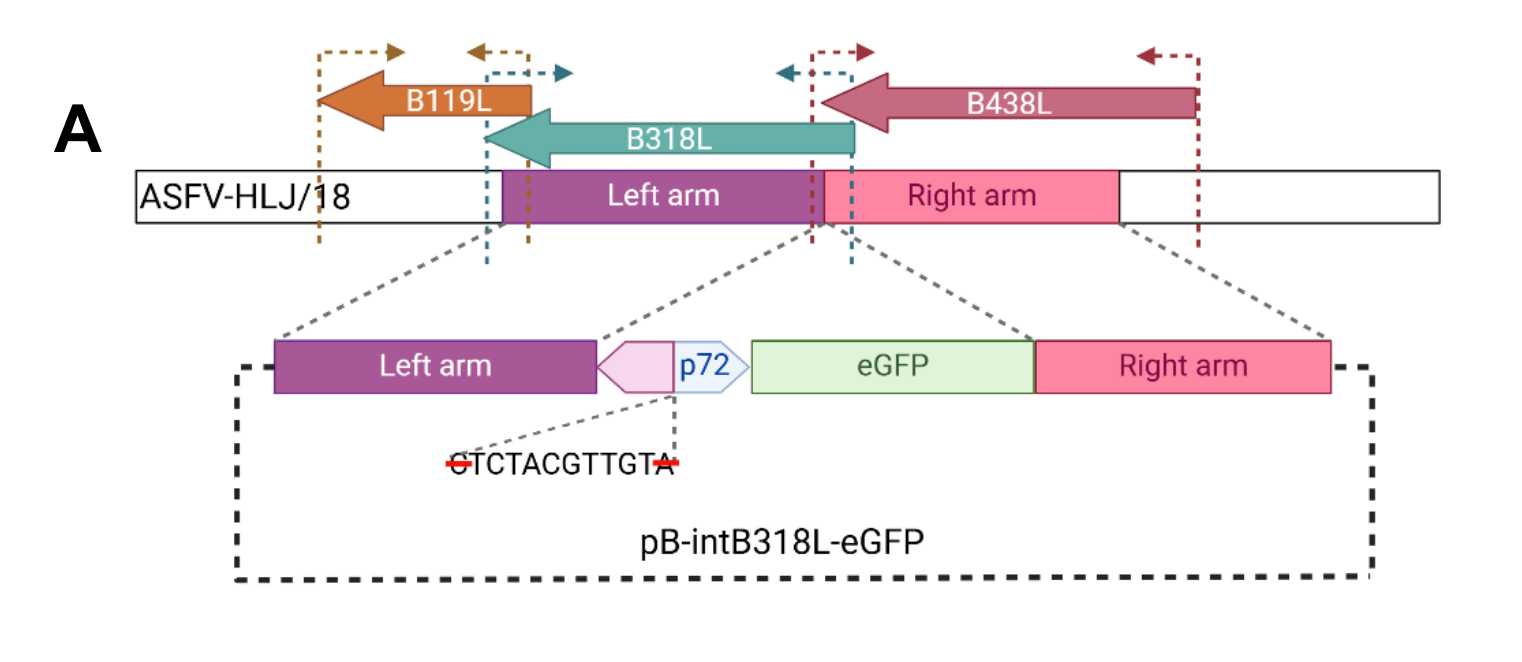

Supplement: S1 Fig — A recombinant African swine fever virus (ASFV) was engineered via homologous recombination in PAMs. The plasmid pBluescript II KS (+) served as the backbone, into which a cassette containing the overlapping sequence of B318L and B438L genes, two LoxP sites, and the enhanced EGFP gene under the control of the ASFV p72 promoter was inserted upstream of the B318L locus. Additionally, the first nucleotide “A” and the tenth nucleotide “C” of the B318L open reading frame (ORF) were deleted to disrupt its coding sequence. Recombinant transfer vector (pB-intB318L-eGFP) containing about 800 bp left homologous arm at the left of 96328 site (A) of ASFV HLJ/18 genome, a reporter gene cassette, followed by about 800 bp right homologous arm at the right of 96239 site (C) of ASFV HLJ/18 genome. (TIF) [file ppat.1013558.s001.tif]

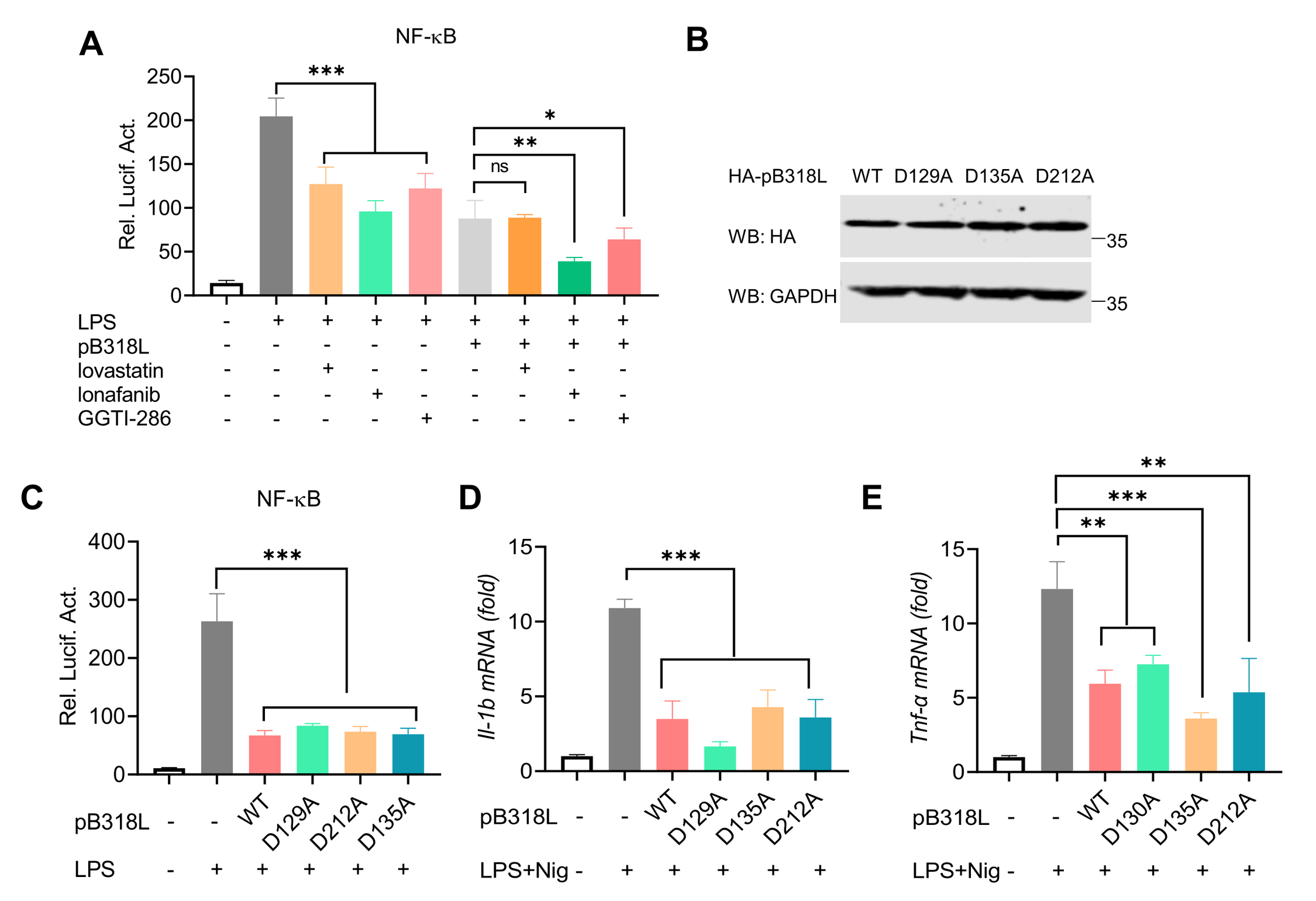

Supplement: S2 Fig — (A) HEK293T cells were transfected with an NF-κB luciferase reporter, a Renilla-TK reporter, and plasmid expressing Flag-pB318L. After 24 h, the cells were treated with LPS for 6 h, and then were treated with Lovastatin, Lonfarnib, GGTI-286 for another 12 h, the luciferase activities were detected. (B) The expression of HA-pB318L enzyme activity mutant plasmid. (C) HEK293T cells were transfected with an NF-κB luciferase reporter, a Renilla-TK reporter, and a plasmid expressing Flag-pB318L-WT or Flag-pB318L-Mut and were treated with LPS for 6 h, the luciferase activities were detected after 24 h. (D-E) HeLa cells were transfected with plasmids expressing Flag-pB318L-WT or Flag-pB318L-Mut, and then treated with LPS for 6 h, the mRNA levels of Il-1b and Tnfα were analyzed by qPCR. (TIF) [file ppat.1013558.s002.tif]

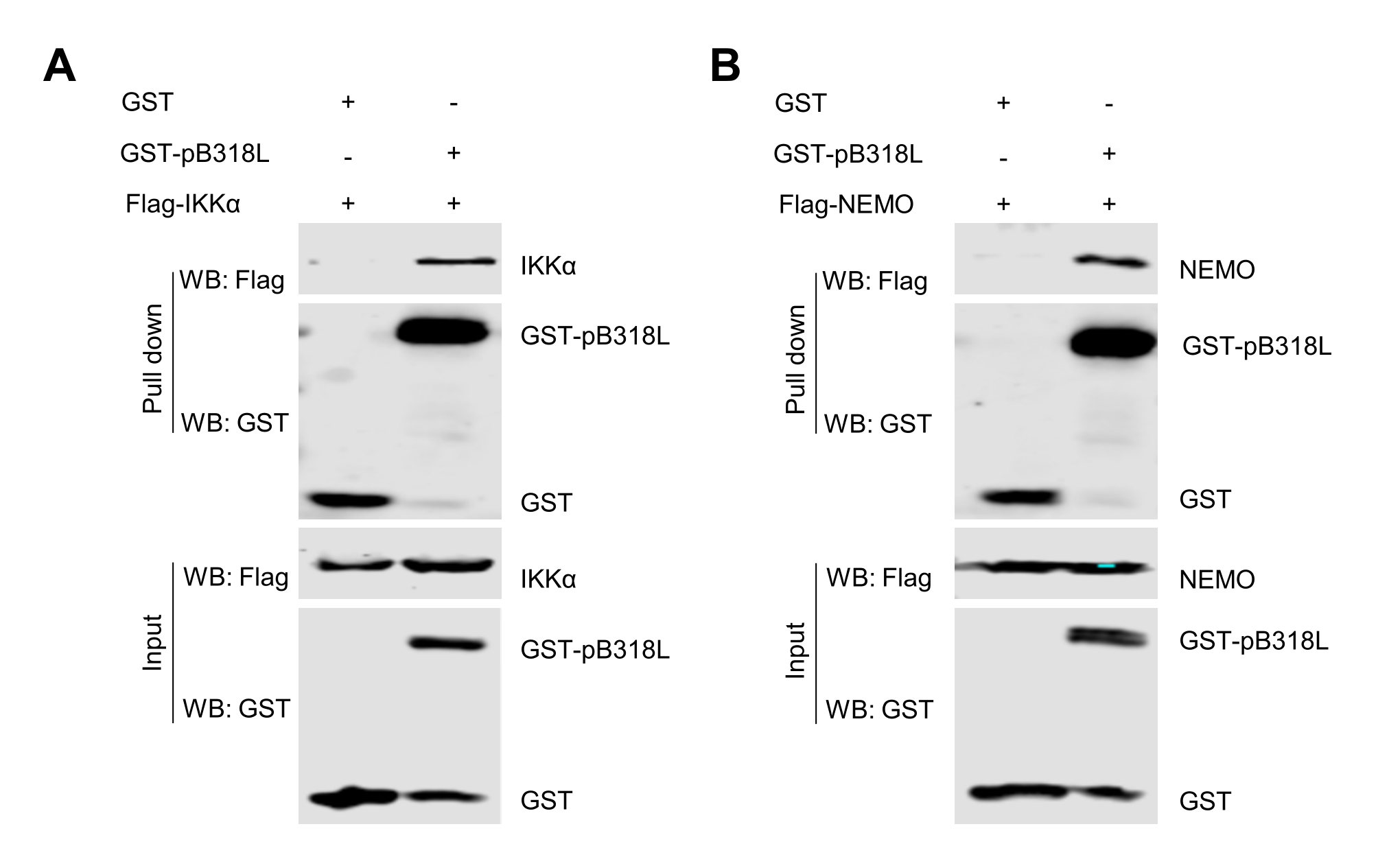

Supplement: S3 Fig — (A) HEK293T cells were transfected with plasmids expressing Flag-IKKα for 24h, Then, cell lysates were immunoprecipitated with GST or GST-pB318L protein and anti-GST beads. Direct interaction between IKKα and ASFV pB318L was detected by GST pull-down assay. (B) HEK293T cells were transfected with plasmids expressing Flag-NEMO for 24h, Then, cell lysates were immunoprecipitated with GST or GST-pB318L protein and anti-GST beads. Direct interaction between NEMO and ASFV pB318L was detected by GST pull-down assay. (TIF) [file ppat.1013558.s003.tif]

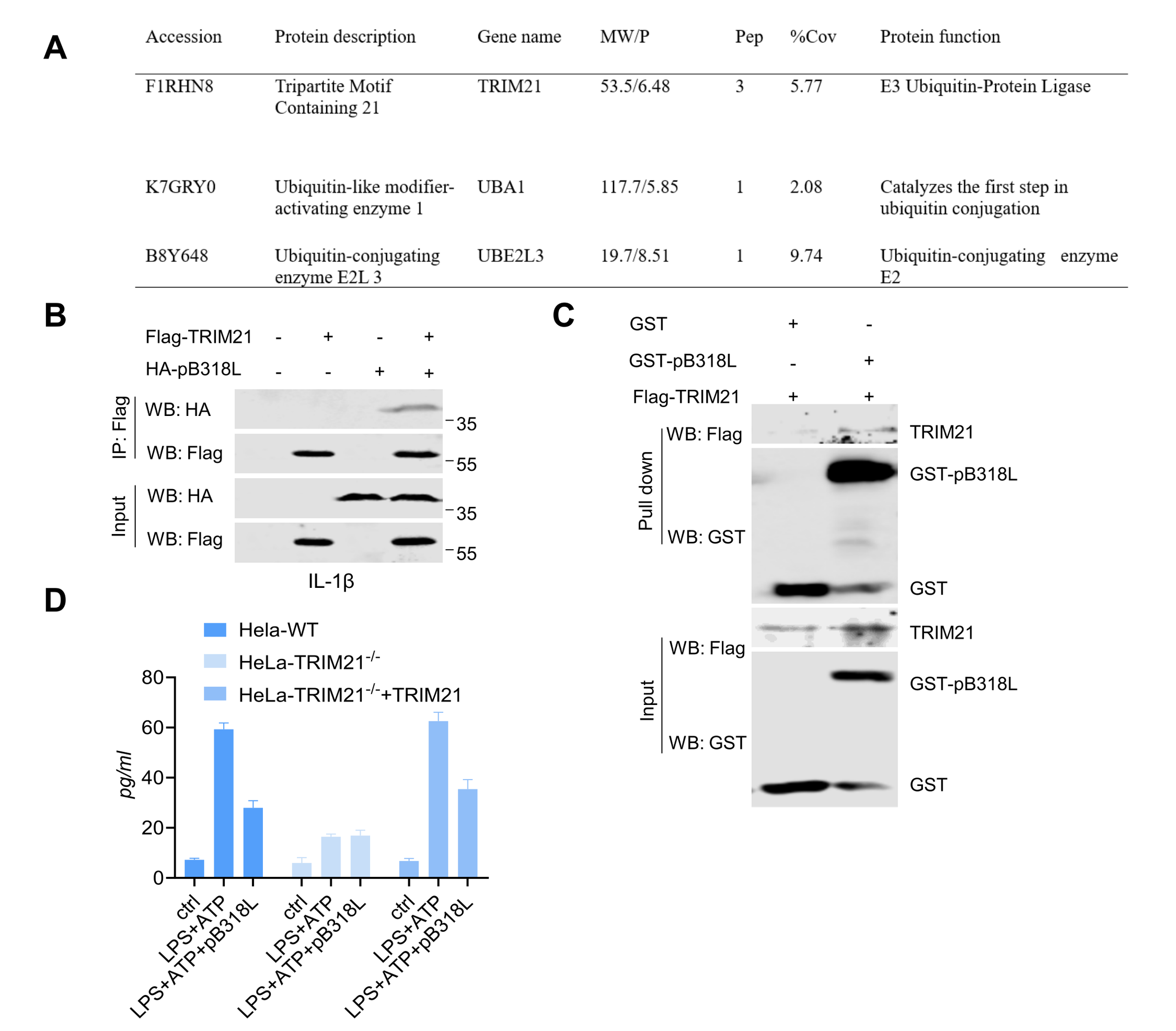

Supplement: S4 Fig — (A) PAMs were infected with ASFV-WT and collected to binding with anti-pB318L antibody, mass spectrometry analysis was performed after SDS-PAGE. TRIM21 was found to interact with pB318L. (B) HEK293T cells were transfected with plasmids expressing HA-pB318L and Flag-TRIM21 for 24 h. Then, cell lysates were incubated with anti-Flag (M2) beads and analyzed through Western blotting. (C) HEK293T cells were transfected with plasmids expressing Flag-TRIM21 for 24h, Then, cell lysates were immunoprecipitated with GST or GST-pB318L protein and anti-GST beads. Direct interaction between TRIM21 and ASFV pB318L was detected by GST pull-down assay. (D) HeLa cells, HeLa deletion of TRIM21 or TRIM21 rescue in HeLa-TRIM21 cells were transfected with plasmids expressing HA-pB318L and treated witn LPS + ATP. Cell supernatants were collected and the protein levels of IL-1β in the supernatants was detected. (TIF) [file ppat.1013558.s004.tif]

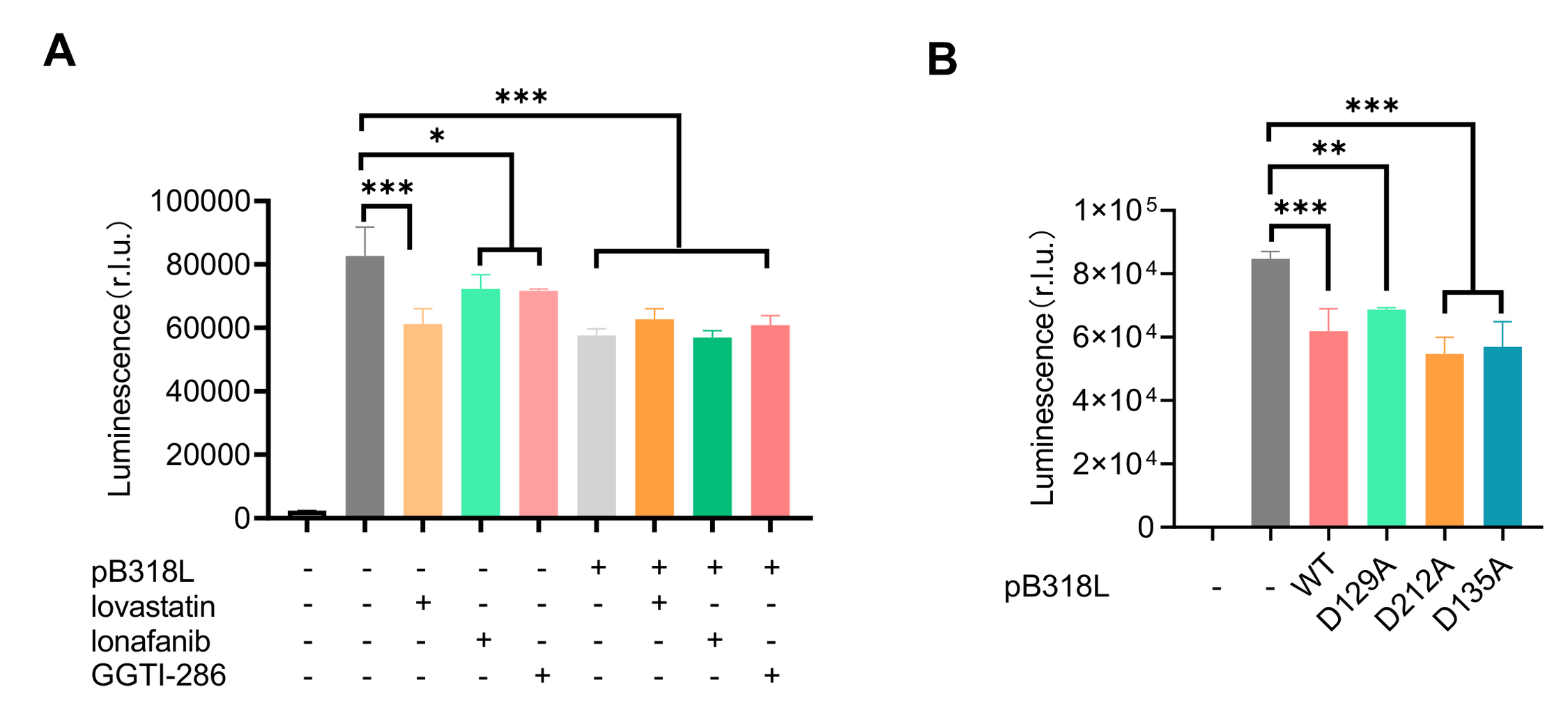

Supplement: S5 Fig — (A) HEK293T cells were transfected with iGLuc-based NLRP3 inflammasome system, and plasmid expressing Flag-pB318L. After 24 h, the cells were treated with Lovastatin, Lonfarnib, GGTI-286 for 12 h, the luciferase activities were detected. (B) HEK293T cells were transfected with iGLuc-based NLRP3 inflammasome system, and plasmids expressing Flag-pB318L-WT or Flag-pB318L-Mut and the luciferase activities were detected after 24 h. (TIF) [file ppat.1013558.s005.tif]

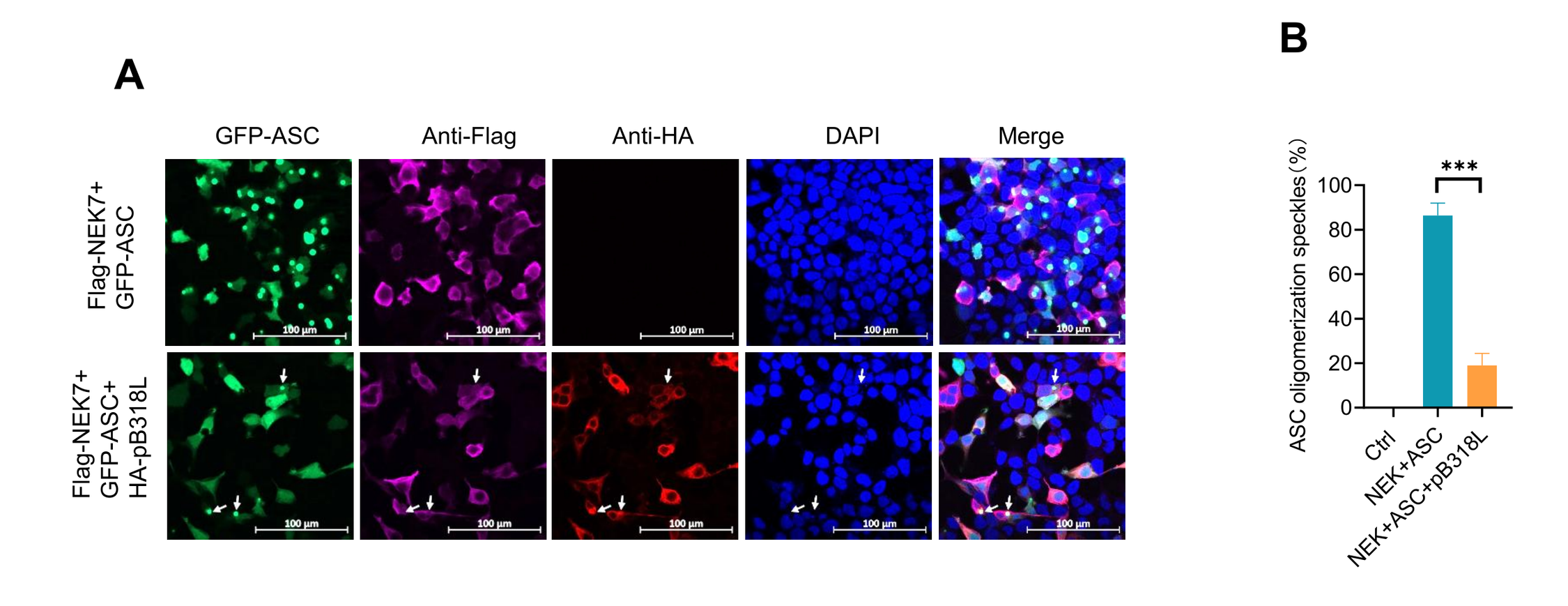

Supplement: S6 Fig — (A) CRL-2843 cells were transfected with plasmids expressing GFP-ASC, Flag-NEK7 and HA-pB318L. The oligomerization of ASC was observed by confocal microscopy. (B) Randomly select three large fields of view, and quantify the percentage of NEK7-induced ASC specks as well as that of NEK7-induced ASC specks under the condition of pB318L co-expression. (TIF) [file ppat.1013558.s006.tif]
