# Supplementary material for: African swine fever virus pB318L suppresses inflammatory response by inhibiting NF-κB activation and NLRP3 inflammasome formation
Source: PLoS Pathog. 2025 Oct 22;21(10):e1013558. doi: 10.1371/journal.ppat.1013558 (PMC12543117; doi:10.1371/journal.ppat.1013558)
Supplement: S1 Table — (DOCX) [file ppat.1013558.s007.docx]

## S1 Table. Primers used for plasmid construction in this study.

| Plasmids | Primers (5'-3') |
| --- | --- |
| pCAGGS-Flag-B318L  -D129A | F1:CCGGGCATGCCTCGAGCTAGCATGTTGCATCTCATCTATATCTCC  R1: TGTCAAAGGAGGGCATATCGGCGATAATCAAAGAAGCTACGTGAAAGTAC  F2: GTACTTTCACGTAGCTTCTTTGATTATCGCCGATATGCCCTCCTTTGACA  R2: ATCCTTGTAATCCATAGATCTTTAGGTCCCCAATGCAACATTT |
| pCAGGS-Flag-B318L  -D135A | F1: CCGGGCATGCCTCGAGCTAGCATGTTGCATCTCATCTATATCTCC  R1: ATTTCGCCGCTTCACATCGTTGGCAAAGGAGGGCATATCGTCGATAATCAAA  F2: TTTGATTATCGACGATATGCCCTCCTTTGCCAACGATGTGAAGCGGCGAAAT  R2: ATCCTTGTAATCCATAGATCTTTAGGTCCCCAATGCAACATTT |
| pCAGGS-Flag-B318L  -D212A  pCAGGS-HA-TLR4 | F1: CCGGGCATGCCTCGAGCTAGCATGTTGCATCTCATCTATATCTCC  R1: TAAAAAAGGGAATGGTTTTTTCTGGAGTGTCTACTAACTGACCGGAACCCG  F2: CGGGTTCCGGTCAGTTAGTAGCCACTCCAGAAAAAACCATTCCCTTTTTTA  R2: ATCCTTGTAATCCATAGATCTTTAGGTCCCCAATGCAACATTT  F: TTCGAGCTCATCGATGGTACCATGATTCCTCGCATCCGCCTG  R: ATTAAGatctgctagctcgagagtgaaggctgttgtatcatg |
| pCAGGS-HA-Myd88 | F: TTCGAGCTCATCGATGGTACCATGGCTGCAGGAGGCTCCGGAGC |
|  | R: ATTAAGatctgctagctcgagGGGCAGGGATAGGGCC |
| pCAGGS-HA-IKKα | F: TTCGAGCTCATCGATGGTACCATGGAGCGGCCCCCGGGGCT |
| pCAGGS-Flag-IKKα-D1  pCAGGS-Flag-IKKα-D2 | R: ATTAAGatctgctagctcgagTCCTGTTAACCAACTCCA  F: CCGGGCATGCCTCGAGCTAGCATGGAGCGGCCCCCGGGGCT  R: ATCCTTGTAATCCATAGATCTAGTATTGTCAGCTTCTTTGATGTTA  F: CCGGGCATGCCTCGAGCTAGCGTCATGTTTATGCAGGGAAA  R: ATCCTTGTAATCCATAGATCTTCCTGTTAACCAACTCCA |
| pCAGGS-HA-NEMO | F: TTCGAGCTCATCGATGGTACCATGAGCAGGACCCCCTGGAA |
|  | R: ATTAAGatctgctagctcgagCTACTCGATACACTCCATGA |
| pCAGGS-HA-NEMO-D1 | F: TTCGAGCTCATCGATGGTACCATGAGCAGGACCCCCTGGAA |
|  | R: ATTAAGatctgctagctcgagATTAAGATCTGCTAGCTCGAG |
| pCAGGS-HA-NEMO-D2 | F: TTCGAGCTCATCGATGGTACCATGAGCAGGACCCCCTGGAA |
|  | R: ATTAAGatctgctagctcgagATTAAGATCTGCTAGCTCGAG |
| pCAGGS-HA-NEMO-D3 | F: TTCGAGCTCATCGATGGTACCGTGCAGGTGGACCAGCTGCGC |
|  | R: ATTAAGatctgctagctcgagCTACTCGATACACTCCATGA |
| pCAGGS-HA-NEMO-D4 | F: TTCGAGCTCATCGATGGTACCCAGCGGGAGGAGAAGGAGTT |
|  | R: ATTAAGatctgctagctcgagCTACTCGATACACTCCATGA |
| pCAGGS-HA-NLRP3-D1 | F: TTCGAGCTCATCGATGGTACCATGAGCATGGCAAGCGTCCG  R: ATTAAGATCTGCTAGCTCGAGCACAGGCTCAGAGTGTTGGT |
| pCAGGS-HA-NLRP3-D2  pCAGGS-HA-NLRP3-D3  pCAGGS-HA-NLRP3-D4  pCAGGS-HA-NLRP3  pCAGGS-HA-ASC  pCAGGS-HA-Caspase-1 | F: TTCGAGCTCATCGATGGTACCCACACGGTAGTATTCCAGGG  R: ATTAAGatctgctagctcgagCCAGTTATTGCTCAGGACTG  F: TTCGAGCTCATCGATGGTACCGAATGGGACAATGCAAATCT  R: ATTAAGatctgctagctcgagCTGGGAAGGCTCAAAGACAA  F: TTCGAGCTCATCGATGGTACCCAGTAGATAGTACATGGCAG  R: ATTAAGatctgctagctcgagCTGGGAAGGCTCAAAGACAA  F: TTCGAGCTCATCGATGGTACCATGAGCATGGCAAGCGTCCG  R: ATTAAGatctgctagctcgagCTGGGAAGGCTCAAAGACAA  F: TTCGAGCTCATCGATGGTACCatggggtgcac gcgtgacgc  R: ATTAAGatctgctagctcgagtcagctctgctccaggtcgg  F: TTCGAGCTCATCGATGGTACCatggccgata aggtgctgaa  R: ATTAAGatctgctagctcgag ttaatgtcctgggaagagat |
|  |  |
