# Supplementary material for: African swine fever virus pB318L suppresses inflammatory response by inhibiting NF-κB activation and NLRP3 inflammasome formation
Source: PLoS Pathog. 2025 Oct 22;21(10):e1013558. doi: 10.1371/journal.ppat.1013558 (PMC12543117; doi:10.1371/journal.ppat.1013558)
Supplement: S2 Table — (DOCX) [file ppat.1013558.s008.docx]

## S2 Table. Primers used for qPCR in this study.

| Plasmids | Primers (5'-3') |
| --- | --- |
| sq-TNF-α | F: ACCACGCTCTTCTGCCTACTGC  R: TCCCTCGGCTTTGACATTGGCTAC |
| sq-IL-6 | F: CTGCTTCTGGTGATGGCTACTG |
|  | R: GGCATCACCTTTGGCATCTT |
| sq-IL-1β | F: CCCAAAAGTTACCCGAAGAGG |
|  | R: TCTGCTTGAGAGGTGCTGATG |
| sq-β-actin | F: TGAGAACAGCTGCATCCACTT |
|  | R: CGAAGGCAGCTCGGAGTT |
| sq-NLRP3 | F: TCGGGGCCAGACAGAAAAAG |
|  | R: CACCTTCTGCCAGTTTGTGC |
| hq-IL-1β | F: GCTCGCCAGTGAAATGATGG |
|  | R: AACACGCAGGACAGGTACAG |
| hq-TNF-α | F: GCCCATGTTGTAGCAAACCC |
|  | R: TGAGGTACAGGCCCTCTGAT |
| hq-IL-6 | F: TGAACTCCTTCTCCACAAGCG |
|  | R: ATTTGTGGTTGGGTCAGGGG |
